# Evaluation of Generative Adversarial Networks for High-Resolution Synthetic Image Generation of Circumpapillary Optical Coherence Tomography Images for Glaucoma

**DOI:** 10.1001/jamaophthalmol.2022.3375

**Published:** 2022-09-01

**Authors:** Ashish Jith Sreejith Kumar, Rachel S. Chong, Jonathan G. Crowston, Jacqueline Chua, Inna Bujor, Rahat Husain, Eranga N. Vithana, Michaël J. A. Girard, Daniel S. W. Ting, Ching-Yu Cheng, Tin Aung, Alina Popa-Cherecheanu, Leopold Schmetterer, Damon Wong

**Affiliations:** 1 Singapore Eye Research Institute, Singapore National Eye Centre, Singapore; 2 Academic Clinical Program, Duke-NUS Medical School, Singapore; 3 SERI-NTU Advanced Ocular Engineering (STANCE), Singapore, Singapore; 4 Institute for Infocomm Research, A*STAR, Singapore; 5 Institute of Molecular and Clinical Ophthalmology, Basel, Switzerland; 6 Department of Ophthalmology, Yong Loo Lin School of Medicine, National University of Singapore, Singapore; 7 Department of Ophthalmology and Optometry, Medical University Vienna, Vienna, Austria; 8 Carol Davila University of Medicine and Pharmacy, Bucharest, Romania; 9 Emergency University Hospital, Department of Ophthalmology, Bucharest, Romania; 10 School of Chemical and Biomedical Engineering, Nanyang Technological University, Singapore; 11 Department of Clinical Pharmacology, Medical University Vienna, Vienna, Austria; 12 Center for Medical Physics and Biomedical Engineering, Medical University Vienna, Vienna, Austria

## Abstract

**Importance:**

Deep learning (DL) networks require large data sets for training, which can be challenging to collect clinically. Generative models could be used to generate large numbers of synthetic optical coherence tomography (OCT) images to train such DL networks for glaucoma detection.

**Objective:**

To assess whether generative models can synthesize circumpapillary optic nerve head OCT images of normal and glaucomatous eyes and determine the usability of synthetic images for training DL models for glaucoma detection.

**Design, Setting, and Participants:**

Progressively growing generative adversarial network models were trained to generate circumpapillary OCT scans. Image gradeability and authenticity were evaluated on a clinical set of 100 real and 100 synthetic images by 2 clinical experts. DL networks for glaucoma detection were trained with real or synthetic images and evaluated on independent internal and external test data sets of 140 and 300 real images, respectively.

**Main Outcomes and Measures:**

Evaluations of the clinical set between the experts were compared. Glaucoma detection performance of the DL networks was assessed using area under the curve (AUC) analysis. Class activation maps provided visualizations of the regions contributing to the respective classifications.

**Results:**

A total of 990 normal and 862 glaucomatous eyes were analyzed. Evaluations of the clinical set were similar for gradeability (expert 1: 92.0%; expert 2: 93.0%) and authenticity (expert 1: 51.8%; expert 2: 51.3%). The best-performing DL network trained on synthetic images had AUC scores of 0.97 (95% CI, 0.95-0.99) on the internal test data set and 0.90 (95% CI, 0.87-0.93) on the external test data set, compared with AUCs of 0.96 (95% CI, 0.94-0.99) on the internal test data set and 0.84 (95% CI, 0.80-0.87) on the external test data set for the network trained with real images. An increase in the AUC for the synthetic DL network was observed with the use of larger synthetic data set sizes. Class activation maps showed that the regions of the synthetic images contributing to glaucoma detection were generally similar to that of real images.

**Conclusions and Relevance:**

DL networks trained with synthetic OCT images for glaucoma detection were comparable with networks trained with real images. These results suggest potential use of generative models in the training of DL networks and as a means of data sharing across institutions without patient information confidentiality issues.

## Key Points

**Question:**

Can realistic circumpapillary optical coherence tomography scans of normal and glaucomatous eyes be generated, and can these be used in the training of a deep learning (DL) glaucoma detection model?

**Findings:**

In this cross-sectional study of 990 normal eyes and 862 glaucomatous eyes, identification of real images from synthetic images by 2 clinicians appeared similar. DL detection models for glaucoma trained exclusively on synthetic images resulted in comparable performance on independent internal and external test sets with a model trained exclusively on real images.

**Meaning:**

Generative models can synthesize realistic optical coherence tomography scans, which can be used to train DL glaucoma detection models and for data sharing.

## Teaser

This cross-sectional study assesses whether generative models can synthesize circumpapillary optic nerve head optical coherence tomography (OCT) images of normal and glaucomatous eyes and determines the usability of synthetic images for training deep learning models for glaucoma detection.

## Introduction

Glaucoma is a progressive eye disease that causes irreversible damage to the retinal ganglion cells,^1^ leading to blindness.^2^ Because of the asymptomatic nature of the disease, loss of sight is often undetected until advanced stages. By 2040, an estimated 111.8 million people^3^ will have glaucoma, which makes earlier detection critical.

Artificial intelligence–based algorithms^4,5,6,7^ have emerged as a potential tool to improve clinical workflows and management^8^ in ophthalmology by detecting patterns associated with conditions such as age-related macular degeneration,^9^ diabetic retinopathy,^5,6^ and glaucoma.^7^ Development of deep learning (DL) models for disease detection requires large amounts of high-quality labeled data. Patient data acquisition is often hindered because of time constraints, privacy concerns, and institutional restrictions. Recently, generative adversarial networks (GANs)^9^ have been extensively used in the artificial intelligence community for the synthesis of artificial images and have emerged as a potential technique to address the challenge of data scarcity in biomedical applications.^10^ In ophthalmic studies, GAN models have been demonstrated in segmentation,^11,12,13,14^ data augmentation,^15,16,17,18,19^ domain transfer,^20,21,22^ image enhancement,^23,24,25,26^ and others.^27^ In data augmentation with GANs, prior studies on synthetic fundus^15,28,29^ and optical coherence tomography (OCT) image generation^16,30^ have largely focused on retinal disorders, which are assessed by the presence of lesions. However, studies on the use of GANs for image synthesis in glaucoma, a disease of the optic nerve characterized by progressive loss, have been limited to fundus photography^18^ and anterior segment OCT imaging^17^ and has not been reported on optic nerve head OCT images (eTable 3 in the Supplement), in which assessment of nerve fiber layer thinning is routinely performed in glaucoma assessment.^31,32^ In this study, we evaluate the use of GANs for the generation of circumpapillary OCT scans for normal and glaucomatous eyes and their potential to train deep neural networks for glaucoma detection.

## Method

### Participants

This retrospective cross-sectional study was conducted from January 2012 to March 2021 and included Chinese participants with glaucoma from 4 clinical studies (2009-2021)^32,33^ and controls from the Singapore Epidemiology of Eye Disease program,^34^ a population-based study (2009-2021) performed at the Singapore Eye Research Institute in Singapore. Additional data from a clinical study of non-Hispanic White participants from the Carol Davila University of Medicine and Pharmacy, Bucharest, Romania (2020-2021), were also included. Both studies were approved by the respective review boards, performed in accordance with the tenets of the Declaration of Helsinki,^35^ and written informed consent was obtained from all participants. Travel allowances were provided to study participants. This study followed the Transparent Reporting of a Multivariable Prediction Model for Individual Prognosis or Diagnosis (TRIPOD) reporting guideline.

Study methodologies were identical for the Singapore and Bucharest sites. Sex and ethnicity were self-reported. Participants were excluded if they exhibited signs of retinal or optic neuropathies other than glaucoma, history of retinal operation or laser treatments, and if they had systemic diseases that might affect the retina or visual field. Glaucoma was based on clinical assessment, having both glaucomatous optic neuropathy and glaucomatous visual field loss. Severity of glaucoma was based on mean deviation (MD) and defined as mild glaucoma (MD, ≥−6 dB), moderate glaucoma (MD, −6.01 to −12.00 dB), or advanced glaucoma (MD, <−12 dB). Normal controls were individuals free from glaucoma and other clinically relevant eye conditions such as macular or vitreoretinal diseases, including epiretinal membrane, diabetic retinopathy, age-related macular degeneration, and other retinopathies that might affect retinal thickness.

### OCT Imaging

Cirrus spectral-domain OCT (Carl Zeiss Meditec) was performed using a 6 × 6 mm^2^ scan protocol centered on the optic nerve head after pupil dilation. Circumpapillary cross-sectional OCT images were obtained with a 3.46-mm diameter circular circle centered on the optic nerve head. Each scan was manually checked and excluded if the signal strength was below 6 or contained substantial movement artefacts.

### Workflow and Development

In this study, we examined the usage of the GANs to produce synthetic images. From participants enrolled at the Singapore study site (a validation set of 84 normal and 84 glaucomatous eyes and an internal test set of 70 normal and 70 glaucomatous eyes) were first randomly selected and set aside to be used during the development of the DL models. Eyes from a participant were not allowed to be in different subsets, and participants were wholly in either the validation or internal test sets. The remaining 990 healthy eyes and 862 glaucomatous eyes were used to develop 2 separate GAN models to generate synthetic normal or glaucoma circumpapillary OCT images. DL models for glaucoma detection were trained using either exclusively real or synthetic images. Detection performance of these DL models were evaluated on the internal test set and on an independent external test set based on the Bucharest data. An overall workflow of the study is provided in eFigure 1 in the Supplement.

### Image Synthesis

For image synthesis, a type of generative model known as progressive GAN (PGGAN) was used.^36^ GANs use adversarial training wherein the probability of the generated images is matched to the real images as the training progresses, using a generator, which takes in a random latent noise vector, and a discriminator, which classifies images as real or synthetic. PGGAN includes incremental addition of layers, which allows for more stable training to generate high-resolution synthetic images. In this study, an output image resolution of 256 × 256 pixels was defined. The development of PGGAN models was done in Pytorch. PGGAN models were trained for approximately 72 hours using a NVIDIA DGX Workstation (Lamda Labs) with 4-T V100 graphic processing units.

Once trained, a latent vector fed as input to the PGGAN model generates a synthetic image and this approach can be extended to produce any number of synthetic images. Two different PGGAN models, 1 for the generation of normal OCT images and 1 for the generation of glaucoma OCT images, were developed separately.

### Clinician Evaluation Methodology

Two hundred OCT images comprising real glaucoma images (n = 50), real normal images (n = 50), synthetic glaucoma images (n = 50), and synthetic normal images (n = 50) were randomly selected and were manually evaluated by 2 expert clinician-scientists with more than 10 years (expert 1: R.S.C.) and 20 years (expert 2: J.G.S.) of experience. The experts were allowed to review the images at a setting and time of their convenience. No prior information regarding the data distribution was given to the clinicians to avoid any bias. The images were prepared on a digital grading form with inputs requested for gradeability and authenticity as follows: (1) gradeability evaluation: the objective of this experiment was to evaluate the quality of the OCT images, including both real and synthetic images, as gradable or nongradable and (2) authenticity evaluation: the 2 clinicians were asked to determine if the OCT images appeared real or synthetic.

### DL Evaluation Methodology

To assess whether synthetic images generated using the PGGAN model for normal and glaucomatous eyes were good enough to be used for training DL algorithms, we compared the diagnostic performance of DL classification models trained only with real images or with only synthetic images. Both DL models used the VGG 11 architecture^37^ and were trained from scratch. Stochastic gradient descent optimizers with learning rate of 0.001, momentum of 0.9, cross entropy loss function and batch size of 16 were used. For training, early stopping with a patience factor of 10 was used and the checkpoint with best performance on the validation data set was saved as the final model.

The DL classification model based on real images was trained using 600 normal eyes and 600 glaucomatous eyes, randomly sampled from the GAN training data. For the DL models trained with synthetic images generated from the GAN models, different data set sizes were used to evaluate the effect of synthetic data set size on DL detection performance. Total data set sizes of 1200, 10 000, 60 000, and 200 000 were generated using the GAN models, with an equal split between the generated synthetic normal and glaucomatous eyes at each data set size. A summary of the DL training data can be found in eTable 2 in the Supplement. Both real and synthetic image-based DL models were tested and evaluated on the unseen real internal and external test images. To avoid any bias, PGGAN model training did not include any of the images from the test sets. A baseline area under the curve (AUC) score was obtained for glaucoma detection using the circumpapillary retinal nerve fiber layer (cpRNFL) thickness.

### Additional Analysis

To evaluate the distribution of the generated synthetic images and real images, a DL-based UNet segmentation^38^ model was used to measure the thickness of the retinal nerve fiber layer (RNFL) layer. Additionally, similarity of the generated images to real images was evaluated using the Fréchet Inception Distance (FID) score,^39^ where a lower FID score indicates a greater similarity. Class activation maps (CAMs)^40^ were generated and visualized as heat maps overlaid on the OCT images to visualize the regions that influenced the classifications of the DL models.

### Statistical Analysis

Evaluation metrics for glaucoma detection were the AUC, sensitivity, and specificity. 95% CIs were generated using bootstrapping clustered at the participant level to adjust for intraeye correlations. Differences in AUC between models were compared by 2-sided Delong tests,^41^ with *P* values less than .05 considered statistically significant. Comparisons were performed between individual models and *P* values were not corrected for multiple comparisons. All the statistical analysis was done using Python and the scikit-learn library.

## Results

### Human Evaluation Results by Clinical Experts

A total of 1016 glaucomatous eyes from 728 Asian Chinese participants and 1144 eyes from 806 Asian Chinese normal controls were enrolled from the Singapore site, while 150 glaucomatous eyes from 77 non-Hispanic White participants and 150 eyes from 83 non-Hispanic White normal controls were enrolled from the Bucharest site. Further characteristics of the participants can be found in eTable 1 in the Supplement.

The results of image gradeability, as evaluated by the 2 clinicians, are presented in Table 1. Image quality as graded by both clinical experts was almost similar: 92.0% for clinical expert 1 and 93.0% for clinical expert 2. However, synthetic images were assessed to have better image quality compared with the real images, which was largely because of the poorer-quality glaucoma images in the real image data set. Furthermore, all the synthetic images generated for normal control eyes using the PGGAN method were gradable. The results of the authenticity evaluation are shown in Table 2, with a similar accuracy score of 51.8% for clinical expert 1 and 51.3% for clinical expert 2, indicating that both clinicians found it difficult to determine the synthetic images from the real images.

**Table 1.** Human Assessment of Gradeability of Circumpapillary Optical Coherence Tomography (OCT) Image Quality^a^

Variable	No. of images	Image quality, No. (%)	
		Clinical expert 1	Clinical expert 2
All	200	184 (92.0)	186 (93.0)
Real	100	88 (88.0)	90 (90.0)
Normal	50	49 (98.0)	46 (92.0)
Glaucoma	50	39 (78.0)	44 (88.0)
Synthetic	100	96 (96.0)	96 (96.0)
Normal	50	50 (100.0)	50 (100.0)
Glaucoma	50	46 (92.0)	46 (92.0)

^a^ To avoid any evaluation bias, image type (real glaucoma/real normal/synthetic glaucoma/synthetic normal) of the 200 images was not revealed to either of the clinicians. Image quality of each of the 200 images was graded by both clinicians. Values indicate the number (%) of images graded as good quality.

**Table 2.** Human Assessment of Authenticity of Real and Synthetic Optical Coherence Tomography (OCT) Images^a^

Variable	Clinical expert 1, %			Clinical expert 2, %		
	Accuracy	Sensitivity	Specificity	Accuracy	Sensitivity	Specificity
All	51.8	51.5	52.0	51.4	48.9	53.5
Normal	41.0	60.0	22.0	55.2	60.4	50.0
Glaucoma	62.0	42.8	82.0	47.6	38.0	57.1

^a^ The ability of clinicians to differentiate whether each of the images are real or synthetic on visual inspection is reported. Accuracy, sensitivity, and specificity scores of discriminations were calculated for all 200 images including 100 normal and 100 glaucoma images.

### DL Classification Model Results

The glaucoma detection performance for the different DL models is presented in Table 3. The mean cpRNFL thicknesses resulted in a baseline AUC of 0.92 for the internal test data set (mean [SD] cpRNFL: normal eyes, 03.0 [14.0] µm; glaucomatous eyes, 71.4 [17.7] µm) and 0.83 for the external test data set (mean [SD] cpRNFL: normal eyes, 103.0 [8.7] µm; glaucomatous eyes, 83.5 [17.7] µm). AUCs for the DL model trained on real images was 0.96 for the internal test data set and 0.84 for the external test data set, whereas the AUCs for the DL models trained on synthetic images ranged from 0.95 to 0.97 for the internal test data set and 0.86 to 0.90 for the external test data set. On the external test data set, the best-performing DL model trained with synthetic images (n = 200 000) resulted in a higher AUC of 0.90 (95% CI, 0.87-0.93; *P* = .002) compared with the DL model (AUC, 0.84 [95% CI, 0.80-0.87]) trained with real images. However, on the internal test data set, AUC scores of the best-performing DL models trained with synthetic images (AUC, 0.97 [95% CI, 0.95-0.99]) were not statistically different (*P* = .49) from the DL model (AUC, 0.96 [95% CI, 0.94- 0.99]) trained with real images.

**Table 3.** Diagnostic Performance of Deep Learning (DL) Classifier Models for Glaucoma Detection

Model	Internal test data set (70 normal eyes, 70 glaucomatous eyes)			External test data set (150 normal eyes, 150 glaucomatous eyes)		
	AUC (95% CI)	Sensitivity at 80% specificity	Sensitivity at 90% specificity	AUC (95% CI)	Sensitivity at 80% specificity	Sensitivity at 90% specificity
cpRNFL (baseline)^a^	0.92 (0.88-0.96)	87.1	75.7	0.83 (0.79-0.87)	61.3	40.7
Real images^b^	0.96 (0.94-0.99)	95.7	88.6	0.84 (0.80-0.87)	74.7	42
Synthetic images, No.^c^						
1200	0.95 (0.92-0.97)	91.4	84.3	0.86 (0.82-0.89)	70.7	53.3
10 000	0.96 (0.94-0.98)	94.3	90.0	0.88 (0.85-0.91)	74.7	58.9
60 000	0.97 (0.94-0.99)	98.6	94.3	0.87 (0.84-0.91)	69.6	56.7
200 000	0.97 (0.95-0.99)	95.7	91.4	0.90 (0.87-0.93)	86.7	69.4

Abbreviations: AUC, area under the curve; cpRNFL, circumpapillary retinal fiber layer.

^a^ Model based on cpRNFL thickness.

^b^ DL model trained using only real images.

^c^ Four DL models trained using synthetic images of varying data set sizes.

Samples of the real and generated images are shown in the Figure (glaucomatous eyes) and in eFigure 2 of the Supplement (normal eyes). To assess the quantitative similarity of the generated synthetic images with the real images, the FID scores were calculated.^24^ For the 4 synthetic data sets with increasing data set sizes, FID score ranged from 15.8 to 16.7 for normal eyes and 12.0 to 15.6 for glaucomatous eyes (eTable 2 in the Supplement). Additionally, CAMs were generated to visually indicate regions that contributed to the respective classifications for the DL networks. It can be observed that the DL models were relatively consistent for both real and synthetic images, with major regions typically located at the temporal and inferior regions of the circumpapillary images, and largely at the RNFL layer, which is consistent with the expected regions of glaucomatous damage^42^ as highlighted in red in the CAM maps (eFigure 4 in the Supplement).

**Figure.**
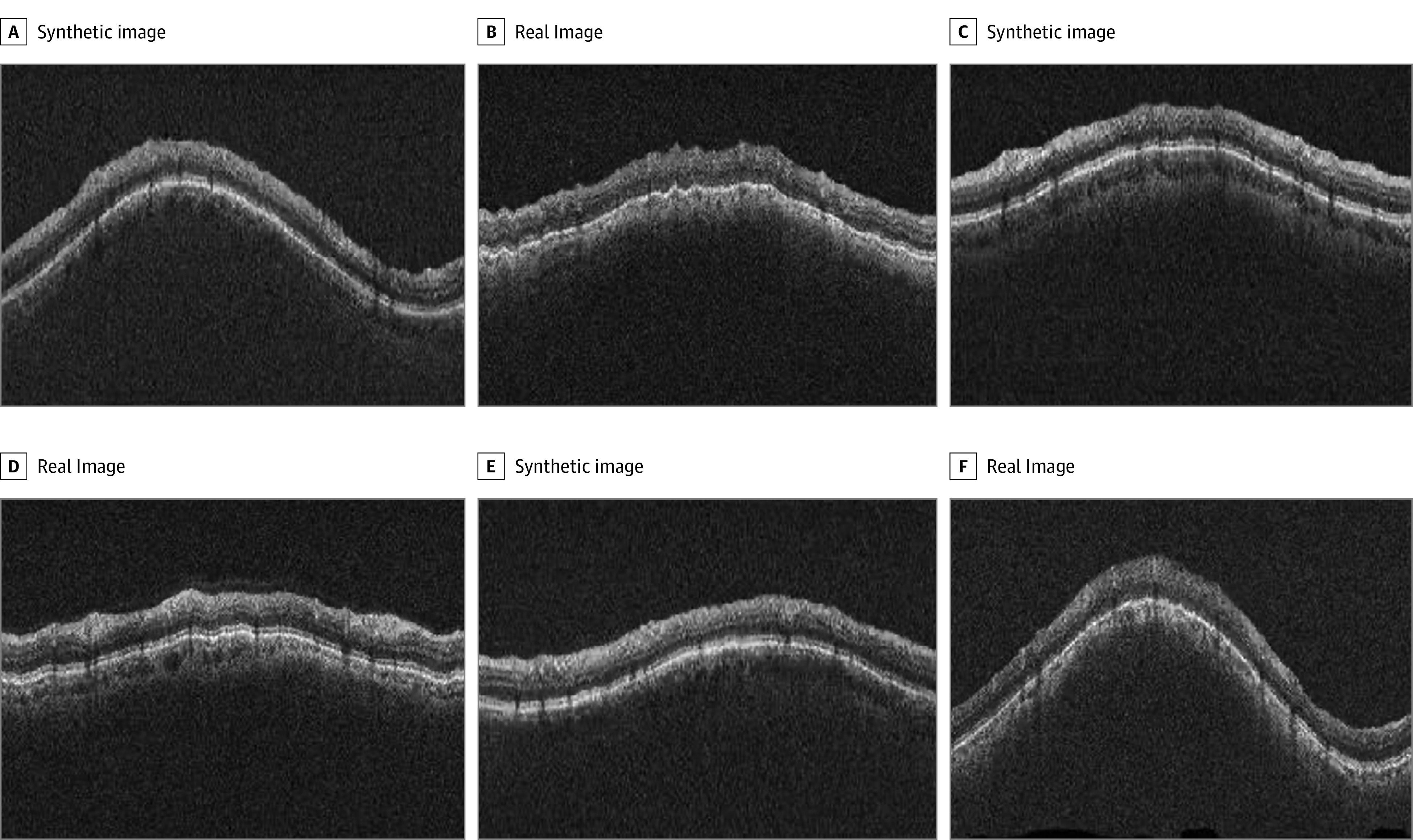
Circumpapillary Optical Coherence Tomography (OCT) Images of Real and Synthetic Glaucomatous Eyes Examples of images of circumpapillary OCT images from real participants and synthetically generated circumpapillary OCT images from the generative adversarial network (GAN) model for glaucomatous eyes. Circumpapillary OCT images of real glaucomatous eyes are located in panels B, D, and E. All other images were synthetically generated from the GAN model for glaucomatous eyes.

## Discussion

In this study, we developed and evaluated GANs for generating high-resolution circumpapillary OCT images for normal and glaucomatous eyes. The results of the quality evaluation demonstrated that synthetic OCT images can be difficult to discern from real images by experienced clinicians. When the synthetic images were used to train a DL model for glaucoma detection, the results showed that the glaucoma detection performance of a model trained with enough synthetic images was comparable with that of a model trained using real data, on both internal test and external test data sets. The development of deep networks for glaucoma detection based on synthetically generated circumpapillary OCT images is a first-of-its-kind approach, and to our knowledge, has not been reported elsewhere. These findings suggest the potentiality of generating realistic-looking synthetic images for the training of deep networks for glaucoma, as well as a possible means of privacy-agnostic data sharing between institutions. In addition, the technology developed for glaucoma in the present work may be translated for DL approaches in rare disease such as hereditary retinal diseases.

Deep models largely depend on the quantity and quality of the input data provided, with a larger number of high-quality data typically associated with improved performance. However, large-scale data acquisition is often resource intensive and involves patient data confidentiality concerns, and an alternative model that efficiently and effectively produces usable data for DL development independent of such constraints is desirable. GANs have seen rapidly increasing interest for its ability to generate realistic synthetic images. Specifically, PGGANs^36^ have been reported in previous ophthalmology-related studies for the generation of fundus images for referrable and nonreferrable age-related macular degeneration^15^ and macula OCT scans for urgent and nonurgent referrals^16^ of eyes with drusen, choroidal neosvacularization, and diabetic macular edema. A recent study also demonstrated the use of synthetic image generation in anterior segment–OCT imaging for angle assessment in glaucoma.^17^ The promising results of the methodology described in these previous works encouraged us to adopt PGGANs for this study. In the gradeability assessment, overall image quality as evaluated by the 2 clinicians was similar. Image quality of the synthetic images outperformed the real images, largely because of the poorer gradeability of real glaucoma images. Clinicians also compared similarly in evaluating the authenticity of the images, which suggests that the generated images were realistic enough to be difficult to discern from real data.

A critical challenge to the development of DL models is the requirement of large data sets. While low-shot approaches could be used as viable strategies to address bias through better data representation with smaller data sets,^43^ generative models were evaluated in this study. We generated synthetic images for normal and glaucomatous eyes, with data set sizes ranging from 1200 to 200 000, and the results of our experiments show that glaucoma detection improved as data set size increased, achieving comparable performance to that of real images in training DL models. The FID scores (range, 12.0-16.7), although moderately higher than values reported on natural images,^36^ provided an indication of the quality of the synthetic images. CAMs shown in eFigure 3 in the Supplement help to identify the regions for classifying the cpRNFL OCT scans as normal or glaucomatous, and it can be observed that for both real and synthetic images, the highlighted regions are consistent, focusing largely on the RNFL layer at the superior and inferior regions. This in general agrees with the patterns of glaucomatous damage, with progressive thinning of the RNFL in eyes with glaucoma.^42,44^

Previous studies^15,16^ reported a decrease in performance when models were trained exclusively on synthetic images and tested on real images. In our study, the DL model trained on 200 000 synthetic images resulted in similar classification performance to that of the DL model trained on 1200 real images on the internal test data set. To evaluate the generalizability of the DL models in our study, we evaluated the performance of DL models on an independent external data set. We observed an increase in performance when the DL model trained on 200 000 synthetic images was tested on the external data set, compared with the cpRNFL baseline model and the glaucoma detection model trained on real images. A possible explanation for the improvement in performance when applied on an external data set could be that extraction of the intrinsic feature space by a GAN followed by usage of feature space representations for task-specific training improves abstraction of generalizable latent space glaucomatous features for training a DL model. However, further experiments are needed to better evaluate this in a more rigorous fashion.

### Limitations

Two separate GAN models for normal and glaucoma images were developed, and future studies could involve the use of conditional GAN-based models to produce synthetic images for specific severities and normal eyes in a single GAN model. Because of the learning of the latent space manifold for GANs, focal changes in the development of glaucoma may be difficult to model. Although models were evaluated on both internal and independent external test sets, the number of test images are relatively small, and studies on larger data sets, with a particular emphasis on nonobvious confounding cases, would be useful. Only circumpapillary images at the optic nerve head from OCTs (Cirrus) were used. While the approach used in this study could also work with data from other OCTs and at the macula in glaucoma, this was not evaluated, nor the effects of combining data from different OCTs. However, it is currently difficult to generate 3-dimensional volumes based on a GAN approach. This is also the reason why we included circumpapillary OCT scans rather than volumetric optic nerve head data. Synthetic images may also contain intrinsic embedded characteristics that are not visually perceivable but may bias discriminatory performance, and such effects may need to be evaluate in studies combining both real and synthetic data for model development.

## Conclusions

Evaluations of the synthetic circumpapillary OCT images by clinical experts and the glaucoma detection models developed with these images suggest that GANs can generate realistic synthetic images that can be used for training deep networks. Synthetic images generated with GANs could potentially facilitate privacy-agnostic data sharing.

## Notes

Supplement. eTable 1. Summary of the Study Participants eTable 2. Summary of Data used for Training Deep Learning Glaucoma Detection Models eTable 3. Summary of Search Terms eFigure 1. Workflow of the study eFigure 2. Real and synthetic circumpapillary OCT images of normal eyes eFigure 3. Distribution of the extracted circumpapillary RNFL thicknesses from the (a) real normal and real glaucoma images used for GAN training, and (b) synthetic 100,000 normal and synthetic 100,000 glaucoma images generated from the normal and glaucoma GAN models respectively eFigure 4. Class Activation Maps of the glaucoma detection models
