# Supplementary material for: Evaluation of Generative Adversarial Networks for High-Resolution Synthetic Image Generation of Circumpapillary Optical Coherence Tomography Images for Glaucoma
Source: JAMA Ophthalmol. 2022 Sep 1;140(10):974–81. doi: 10.1001/jamaophthalmol.2022.3375 (PMC9437828; doi:10.1001/jamaophthalmol.2022.3375)
Supplement: Supplement. — eTable 1. Summary of the Study Participants eTable 2. Summary of Data used for Training Deep Learning Glaucoma Detection Models eTable 3. Summary of Search Terms eFigure 1. Workflow of the study eFigure 2. Real and synthetic circumpapillary OCT images of normal eyes eFigure 3. Distribution of the extracted circumpapillary RNFL thicknesses from the (a) real normal and real glaucoma images used for GAN training, and (b) synthetic 100,000 normal and synthetic 100,000 glaucoma images generated from the normal and glaucoma GAN models respectively eFigure 4. Class Activation Maps of the glaucoma detection models [file jamaophthalmol-e223375-s001.pdf]

Supplemental Online Content

Kumar AJS, Chong RS, Crowston JG, et al. Evaluation of generative adversarial networks for high-resolution synthetic image generation of circumpapillary optical coherence tomography images for glaucoma. *JAMA Ophthalmol*. Published online September 1, 2022. doi:10.1001/jamaophthalmol.2022.3375

eTable 1. Summary of the Study Participants
eTable 2. Summary of Data used for Training Deep Learning Glaucoma Detection Models
eTable 3. Summary of Search Terms
eFigure 1. Workflow of the study
eFigure 2. Real and synthetic circumpapillary OCT images of normal eyes
eFigure 3. Distribution of the extracted circumpapillary RNFL thicknesses from the (a) real normal and real glaucoma images used for GAN training, and (b) synthetic 100,000 normal and synthetic 100,000 glaucoma images generated from the normal and glaucoma GAN models respectively
eFigure 4. Class Activation Maps of the glaucoma detection models

This supplemental material has been provided by the authors to give readers additional information about their work.

© 2022 Kumar AJS et al. *JAMA Ophthalmology.*

eTable 1. Summary of the Study Participants

**Study Site**	Singapore		Bucharest	
**Ethnicity**	Asian Chinese		Non-Hispanic White	
**Participants**	Healthy	Glaucoma	Healthy	Glaucoma
**Eyes / Participants**	1144 / 806	1144 / 728	150 / 83	150 / 77
**Sex, male**	418 (51.9%)	485 (66.6%)	55 (66.3%)	50 (64.9%)
**Age, years**	54.1±7.1	65.1±9.1	39.1±11.1	64.7±11.1
**Refractive Error (D)**	-0.9±2.3	0.6±3.1	0.0±2.0	0.3±1.1
**Average RNFL thickness(µm)**	103.8±11.9	76.6±14.5	103.1 ±8.7	83.6 ± 17.7
**Mean Deviation (dB)**	-	-6.5±5.6	-	-4.7±5.7
**Mild**	-	607 (53.1)	-	108 (72.0)
**Moderate**	-	254 (22.2)	-	25 (16.7)
**Advanced**	-	155 (13.5)	-	17 (11.3)

D = Diopters; RNFL = Retinal Nerve Fiber Thickness; dB = Decibels. Sex was self-reported. Refractive error was provided by the spherical equivalent, which was calculated as the sum of the spherical value with half the cylinder value. Data presented as mean±standard deviation or number (%), as appropriate.

© 2022 Kumar AJS et al. *JAMA Ophthalmology.*

eTable 2. Summary of Data used for Training Deep Learning Glaucoma Detection Models

Image Type	Real		Synthetic		Synthetic		Synthetic		Synthetic	
**Class**	**Normal**	**Glaucoma**	**Normal**	**Glaucoma**	**Healthy**	**Glaucoma**	**Healthy**	**Glaucoma**	**Healthy**	**Glaucoma**
**N**	600	600	600	600	5000	5000	30000	30000	100000	100000
**FID**	-	-	16.78	15.64	16.39	12.51	15.92	12.24	15.84	12.02
**Mean RNFL (µm)**	102.7±10.5	76.2±14.3	104.1±11.6	77.9±13.4	104.7±11.4	78.1±13.2	104.5±11.6	78.2±13.3	104.7±11.6	78.2±13.4
**Min RNFL (µm)**	62.8	34.1	71.6	43.3	55.9	38.14	49.1	33.3	50.9	32.3
**Max RNFL (µm)**	134.5	119.9	139.2	116.3	140.1	131.35	143.3	135.3	147.2	134.9
**N_{2SD}**	26 (4.3%)	29 (4.8%)	33 (5.5%)	27 (4.5%)	248 (4.1%)	208 (3.5%)	1487 (2.5%)	1304 (2.2%)	4915 (8.2%)	4242 (7.1%)

FID = Frechet Inception Distance; RNFL = Retinal Nerve Fiber Thickness; N_{2SD} = Number of images more than 2 standard deviations from the mean.

Data presented as mean ± standard deviation or number (%), as appropriate.

© 2022 Kumar AJS et al. *JAMA Ophthalmology.*

eTable 3. Summary of Search Terms

Databases: Google Scholar, PubMed, Semantic Scholar, arXiv

Topic	Search Items
GANs for synthetic Images of optic nerve OCT in glaucoma	GAN, generative adversarial network, synthetic, OCT, optic nerve head, optic disc, glaucoma
GANs for synthetic Images of OCTs for retinal disease in the macula	GAN, generative adversarial network, synthetic, OCT, macula, retina, retinal disease
GANs in ophthalmology	GAN, generative adversarial network, synthesis, ophthalmology, OCT, fundus

© 2022 Kumar AJS et al. *JAMA Ophthalmology.*

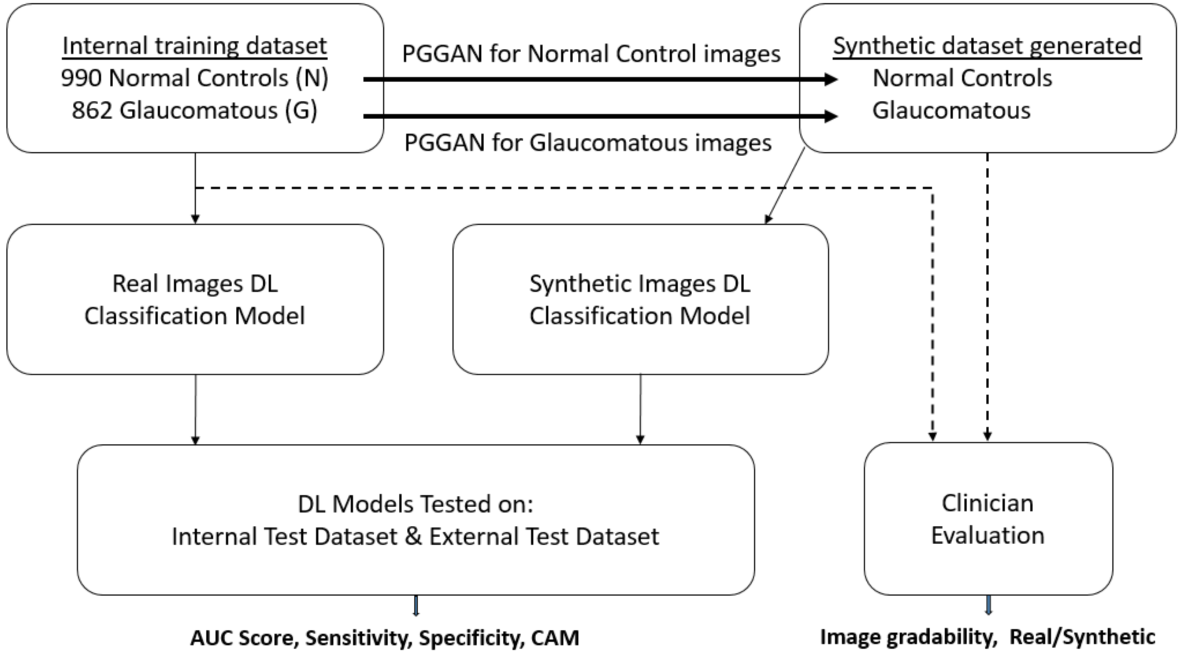

eFigure 1. Workflow of the study. Two separate PGGAN models were developed using real images to generate normal and glaucoma circumpapillary images. The generated images were evaluated by clinicians for gradeability and authenticity, and also used to train deep learning-based glaucoma detection models. Performance of the DL models were evaluated on internal test set and on an independent test set of data collected at a different study site. Results were compared with glaucoma detection based on global retinal nerve fiber layer thickness and a DL model trained on real data. (PGGAN = Progressively Growing Generative Adversarial Model; DL = Deep Learning; AUC: Area Under the Curve; CAM: Class Activation Map)

© 2022 Kumar AJS et al. *JAMA Ophthalmology.*

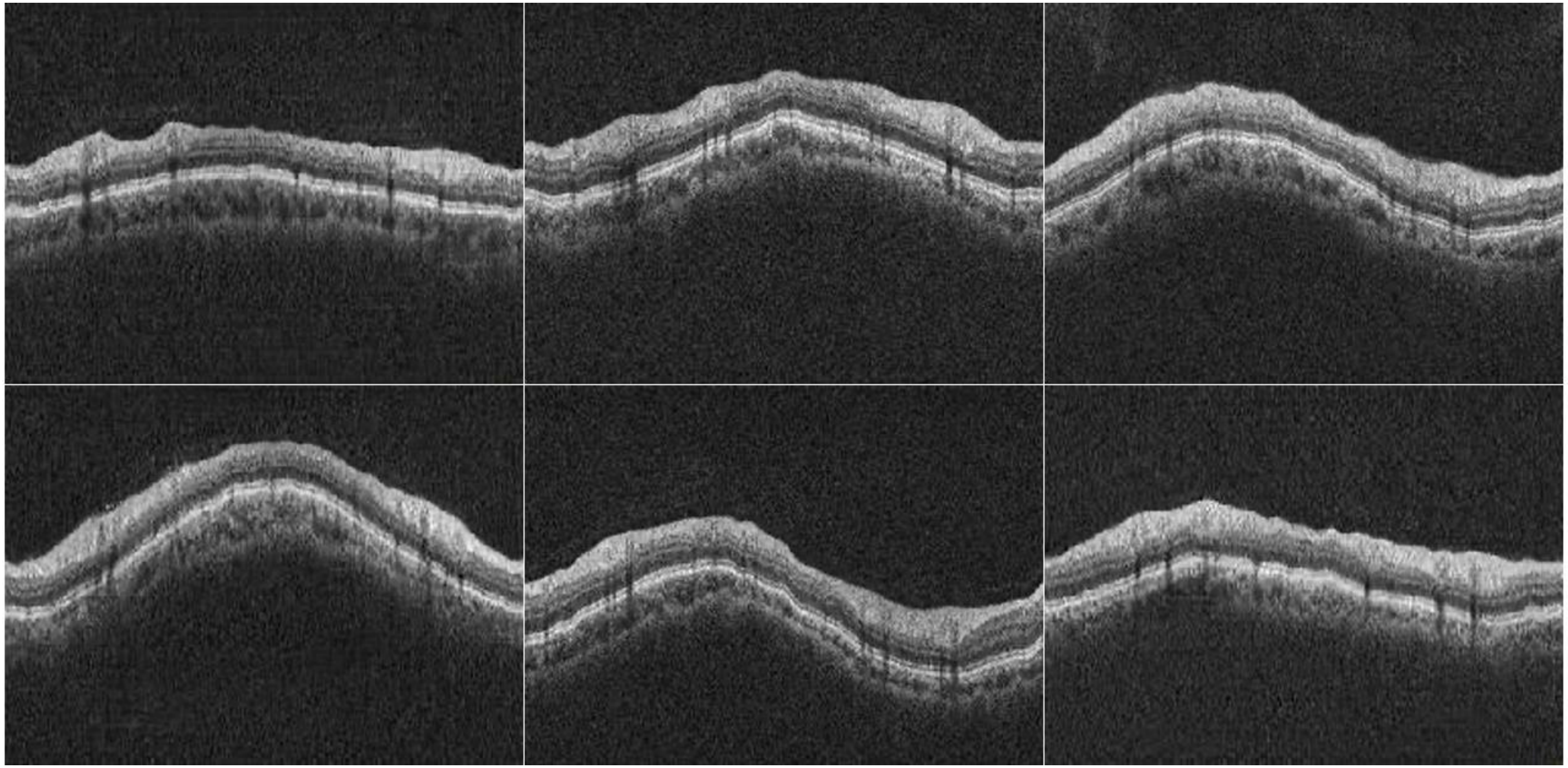

eFigure 2. Real and synthetic circumpapillary OCT images of normal eyes.
Circumpapillary OCT images of real normal eyes are located at first row, left; first row, centre and second row, right. All other images were synthetically generated from the GAN model for normal eyes.

© 2022 Kumar AJS et al. *JAMA Ophthalmology.*

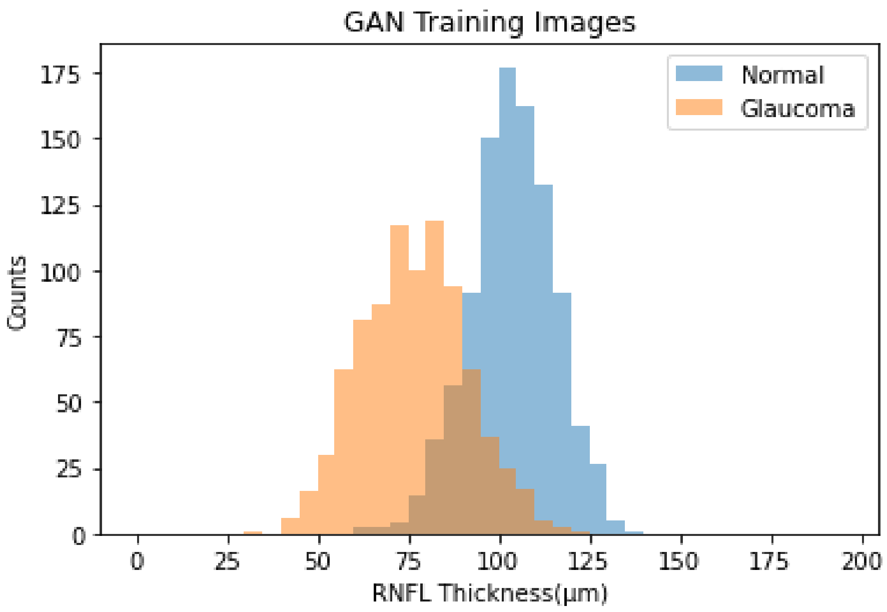

(a)

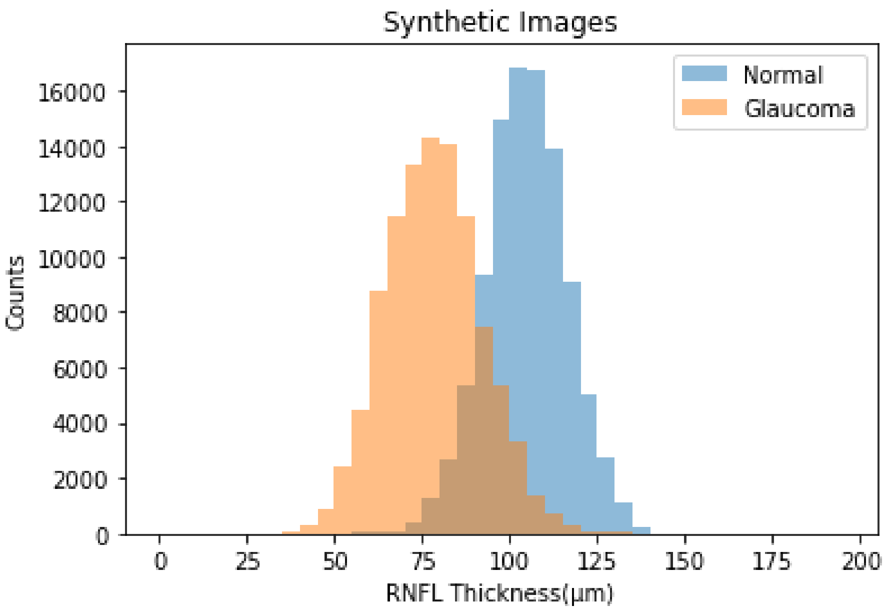

(b)

eFigure 3. Distribution of the extracted circumpapillary RNFL thicknesses from the (a) real normal and real glaucoma images used for GAN training, and (b) synthetic 100,000 normal and synthetic 100,000 glaucoma images generated from the normal and glaucoma GAN models respectively.

© 2022 Kumar AJS et al. *JAMA Ophthalmology.*

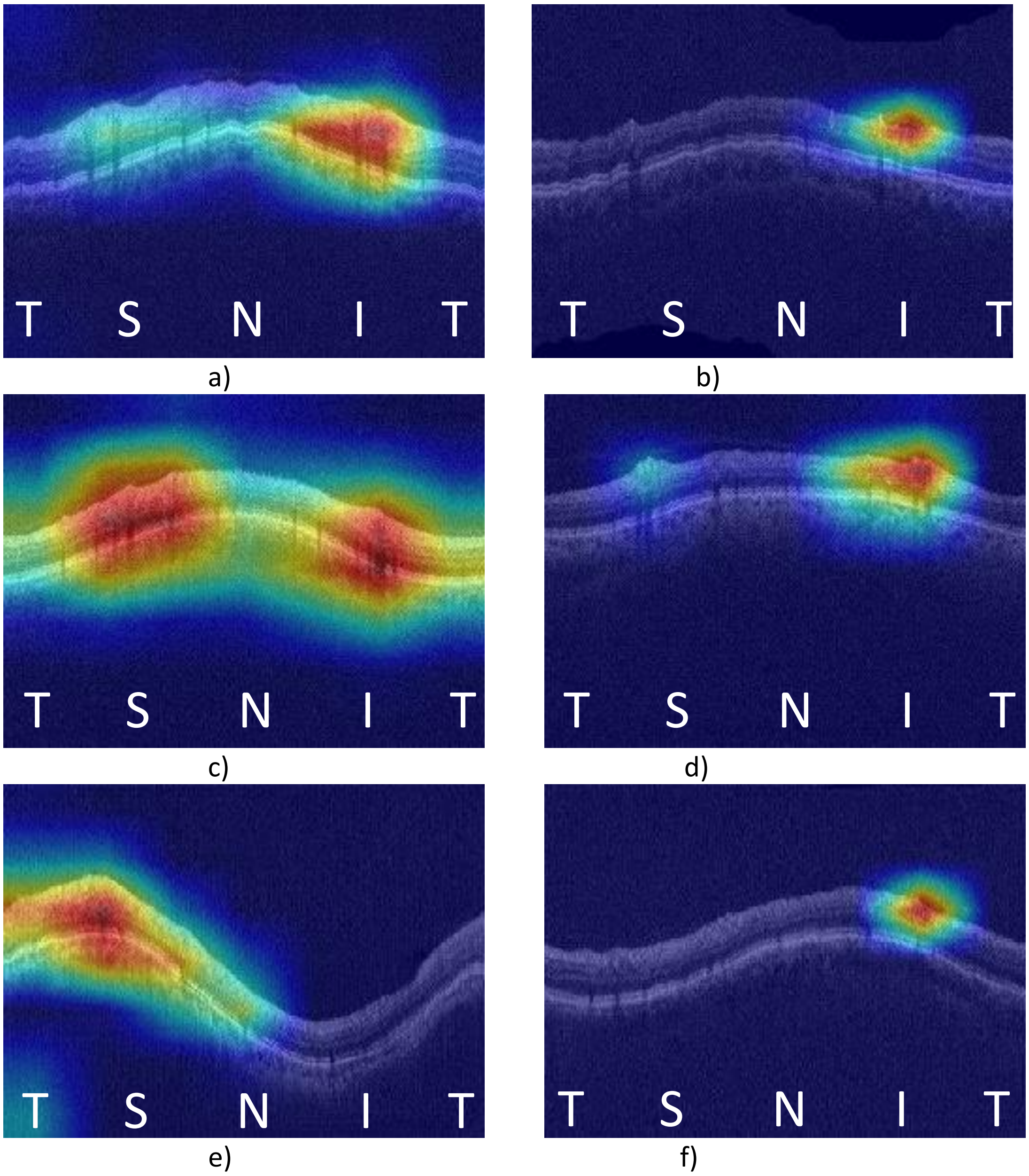

eFigure 4. Class Activation Maps of the glaucoma detection models. Class activation map samples for validating the performance of synthetic images classifier model on the internal test dataset (1st row), external test dataset (2nd row), and synthetic images test dataset (3rd row). 1st column is for normal controls and 2nd column is for glaucomatous eyes. Red highlighted regions correspond to the pixels that mostly contributed to the classification of images as normal controls or glaucomatous eyes. (T = Temporal, S = Superior, N = Nasal, I = Inferior)

© 2022 Kumar AJS et al. *JAMA Ophthalmology.*
